# Distinct Regulation of the Expression of Satellite DNAs in the Beetle *Tribolium castaneum*

**DOI:** 10.3390/ijms22010296

**Published:** 2020-12-30

**Authors:** Antonio Sermek, Isidoro Feliciello, Đurđica Ugarković

**Affiliations:** 1 Department of Molecular Biology, Ruđer Bošković Institute, Bijenička 54, HR-10000 Zagreb, Croatia; Antonio.Sermek@irb.hr (A.S.); ifelicie@unina.it (I.F.); 2 Dipartimento di Medicina Clinica e Chirurgia, Universita’ degli Studi di Napoli Federico II, Via Pansini 5, I-80131 Napoli, Italy

**Keywords:** satellite DNA, siRNA, piRNA, embryogenesis, heat stress, heterochromatin, euchromatin, histone modification

## Abstract

In the flour beetle, *Tribolium castaneum* (peri)centromeric heterochromatin is mainly composed of a major satellite DNA TCAST1 interspersed with minor satellites. With the exception of heterochromatin, clustered satellite repeats are found dispersed within euchromatin. In order to uncover a possible satellite DNA function within the beetle genome, we analysed the expression of the major TCAST1 and a minor TCAST2 satellite during the development and upon heat stress. The results reveal that TCAST1 transcription was strongly induced at specific embryonic stages and upon heat stress, while TCAST2 transcription is stable during both processes. TCAST1 transcripts are processed preferentially into piRNAs during embryogenesis and into siRNAs during later development, contrary to TCAST2 transcripts, which are processed exclusively into piRNAs. In addition, increased TCAST1 expression upon heat stress is accompanied by the enrichment of the silent histone mark H3K9me3 on the major satellite, while the H3K9me3 level at TCAST2 remains unchanged. The transcription of the two satellites is proposed to be affected by the chromatin state: heterochromatin and euchromatin, which are assumed to be the prevalent sources of TCAST1 and TCAST2 transcripts, respectively. In addition, distinct regulation of the expression might be related to diverse roles that major and minor satellite RNAs play during the development and stress response.

## 1. Introduction

Nuclear small RNAs such as small interfering RNAs (siRNAs) and PIWI-interacting RNAs (piRNAs) are involved in chromatin modification by directing the installation of the heterochromatin mark H3K9me3 at their homologous genomic targets, which leads to transcriptional silencing [1,2]. siRNAs are 21–22 nt long and are derived from repetitive sequences, in particular from satellite DNAs, and their role in heterochromatin establishment has been demonstrated in the yeast *Schizosaccharomyces pombe*, plants and insects [3,4,5]. However, piRNAs, which are ~26–30 nt long, mostly derive from transposon sequences and their major role is to repress transposon activity at both the transcriptional and translational level [6]. piRNAs are expressed in the animal germ line with the primary aim of protecting genome integrity, but they are also present in the somatic tissues of many arthropods [7]. siRNAs and piRNAs differ in their biogenesis, while siRNA is derived from long double stranded transcripts processed by the endonuclease Dicer [8], piRNAs are derived from long single-stranded precursors, which are processed by a unique set of proteins [9,10]. It is interesting that the presence of the H3K9me3 mark at genomic regions giving rise to piRNAs and siRNAs is necessary for their biogenesis [2,11]. Apart from the common H3K9me3 mark, piRNA and siRNA loci require the presence of piRNA and siRNA locus-specific proteins, which modulate transcription initiation, termination and processing of long primary transcripts [6,12].

Satellite DNAs are tandemly repeated sequences located in constitutive heterochromatin usually positioned at pericentromeric and centromeric regions in many eukaryotes [13]. In the flour beetle, *Tribolium castaneum* large blocks of (peri)centromeric heterochromatin on all chromosomes are mostly comprised of the major satellite DNA TCAST1, which makes almost 35% of the genome [14,15]. Apart from TCAST1, numerous other repetitive DNAs are present within the *T. castaneum* genome, some exhibit satellite DNA-like tandem organization and are considered minor, low copy number satellites, while some have transposon-like dispersed types of organization [16,17,18]. While the *T. castaneum* genome has been sequenced, large regions of (peri)centromeric heterochromatin, where most of the repetitive sequences are located, remain unassembled [16]. Analysis of the assembled genome reveals repeats of different satellite DNA and other repetitive families clustered within euchromatin, often in terminal chromosome regions [18,19]. Genomic mapping of somatic transposon-derived piRNAs revealed an overlap with the euchromatic repeat clusters, while most transposon-derived piRNA clusters are found within unmapped scaffolds, indicating their localization within pericentromeric heterochromatin [20].

While preferentially embedded in constitutive heterochromatin, which is considered transcriptionally inert, the transcription of satellite DNAs has been reported in many species and is often modulated during development, cell differentiation or in response to environmental stimuli [13,21]. Transcription of the major *T. castaneum* satellite DNA, TCAST1, depends on RNA polymerase II initiated from internal promoters and the resulting long primary transcripts are processed into small RNAs, which have not been characterized until now [22]. In addition, TCAST1 transcription is strongly induced by heat stress (HS) and is accompanied by an increase in repressive histone marks H3K9me2/3 at TCAST1 repeats, resulting in the transient suppression of TCAST1-associated gene expression [23]. It is not known whether other *T. castaneum* satellites exhibit features similar to TCAST1 with respect to a burst of transcripts during embryogenesis and upon HS, followed by their processing into small RNAs, and if their transcripts can affect the H3K9me3 level at homologous satellite repeats upon HS. Here, using high-throughput sequencing of long and small RNAs combined with qRT-PCR, we analyse and compare the transcription and processing of both the major and minor *T. castaneum* satellite DNAs TCAST1 and TCAST2, during development, in particular embryogenesis, as well as in response to HS. The minor TCAST2 satellite comprises 0.5% of genomic DNA and approximately 70% of these repeats are in pericentromeric heterochromatin on all chromosomes, while the remaining repeats are within euchromatic repeat clusters [24].

Our results reveal the difference in the transcription dynamics of the two satellites during development and upon heat stress. While TCAST1 transcription is strongly induced at specific embryonic stages as well as upon heat stress, TCAST2 transcription is not significantly affected during both processes. Consequently, the levels of the silent histone mark H3K9me3 at TCAST2 repeats is not influenced by HS. The variation in transcription dynamics is proposed to be related to chromatin state, which is either euchromatin or heterochromatin. The major TCAST1 transcripts are differentially processed during development, preferentially into piRNAs during embryogenesis and almost exclusively into siRNAs at later developmental stages, suggesting that TCAST1 piRNA production occurs in only the germ line. In contrast, TCAST2 transcripts are processed exclusively into piRNA throughout all developmental stages. Our findings reveal a distinct regulation of expression of different satellite DNAs during the development and upon environmental stress and we discuss the diverse roles that their transcripts might play during these processes, particularly regarding heterochromatin establishment and maintenance, as well as the modulation of nearby genes expression.

## 2. Results

### 2.1. Transcription of TCAST1 and TCAST2 Satellite DNA during Development and Upon Heat Stress

**Figure 1:**
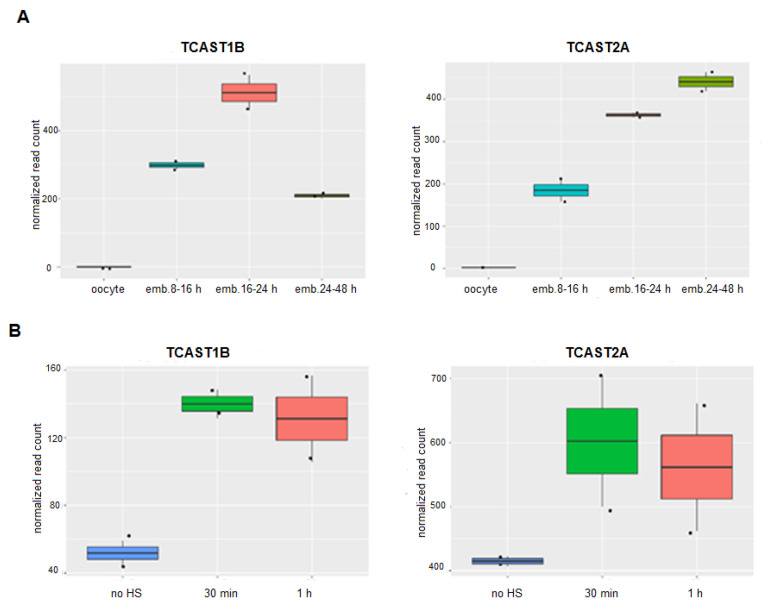
Transcription dynamics of satellite DNAs TCAST1 and TCAST2 during phases of embryogenesis (**A**) and in adults subjected to heat stress, after 30 min and 1 h of recovery as well as in control adults (**B**), obtained from RNASeq data. The program kallisto was used for the quantification of satellite transcripts and normalization of reads was performed by the DESeq2 package. Each sample is based on two biological replicates and error bars indicate the standard deviations.

Transcription of the major TCAST1 and the minor TCAST2 satellite DNA during embryonic development was analysed using long RNAseq libraries from *T. castaneum* oocytes, transcriptionally active early blastoderm (8–16 h), differentiating blastoderm and gastrulation (16–24 h) and germand elongation and full extension period (24–48 h), retrieved from GSE63770 [25]. TCAST1 and TCAST2 satellites are composed of different subfamilies [15,24] and we followed the transcription of the most highly represented subfamily of each satellite: TCAST1B and TCAST2A, respectively. Within the oocytes, no transcripts of either TCAST1B or TCAST2A satellites were detected, which is in accordance with the absence of transcription at this stage (Figure 1A). The transcription of both satellites is observed within the early blastoderm and continues to increase during differentiation of the blastoderm and gastrulation phase (16–24 h). During this period the level of TCAST1B RNA reaches a peak and drops significantly during the next period (24–48 h). In contrast, the level of TCAST2A RNA does not drop during germand elongation and the full extension period (24–48 h), but is slightly increased (Figure 1A).

Transcription of the major TCAST1 satellite DNA throughout the developmental stages of larvae, pupae and adults is stable, proceeding equally from both DNA strands, but it is strongly induced in adult beetles subjected to short (4 h) or long (24 h) heat stress, as shown previously [22,23]. An increase of TCAST1 RNA levels is observed immediately after HS and remains upregulated for 2–3 h during the recovery period, while transcription of the minor TCAST2 satellite DNA is not affected by HS, except for a slight increase detected at 30 min of recovery period [18]. To confirm the previous results obtained by RT-qPCR, we performed sequencing of the total RNA isolated from *T. castaneum* adults used as a control, as well as from adults subjected to a long HS treatment of 24 h at 40 °C, followed by a recovery period of 30 min and 1 h, respectively. Within the obtained data sets, we analysed the level of TCAST1B and TCAST2A satellite transcripts and compared their amount before and after HS. The results calculated by the DESeq2 package reveal a significant increase in the transcription of TCAST1B, 2.79x (*p* = 0.0010) at 30 min and 2.55x (*p* = 0.0022) at 1 h of recovery period, respectively, relative to the control (Figure 1B), which is in accordance with the results obtained previously by RT-qPCR. For the TCAST2A satellite, a slight but statistically insignificant increase in transcription was observed at 30 min (1.44x, *p* = 0.1311) and 1 h (1.35x, *p* = 0.2212) of recovery period, respectively (Figure 1B), revealing no significant influence of HS on the TCAST2A transcription.

It is interesting that, although the two satellites differ significantly in the copy number, based on the normalized number of reads (Figure 1), their transcription level is comparable during embryogenesis or is even higher for TCAST2 in adults, indicating the absence of a positive correlation between the level of transcripts and satellite copy number.

### 2.2. Small TCAST1 RNAs during Development and Upon Heat Stress

The profiles of TCAST1 and TCAST2 satellite-derived small RNAs during embryogenesis were analysed using small RNAseq libraries from *T. castaneum* oocytes, early embryos before the onset of zygotic transcription (0–5 h), transcriptionally active early blastoderm (8–16 h), differentiating blastoderm (16–20 h), gastrulation (20–24 h), germband elongation (24–34 h), fully-extended germband (34–48 h), and late stage development through hatching (2–6 days), taken from GSE63770 [25]. In addition, profiles of TCAST1 and TCAST2 small RNAs were analysed using small RNAseq libraries of *T. castaneum* adults kept at normal temperature (25 °C), as well as of adults subjected to heat stress for 24 h at 40 °C, followed by a recovery period of 1 h at 25 °C.

**Figure 2:**
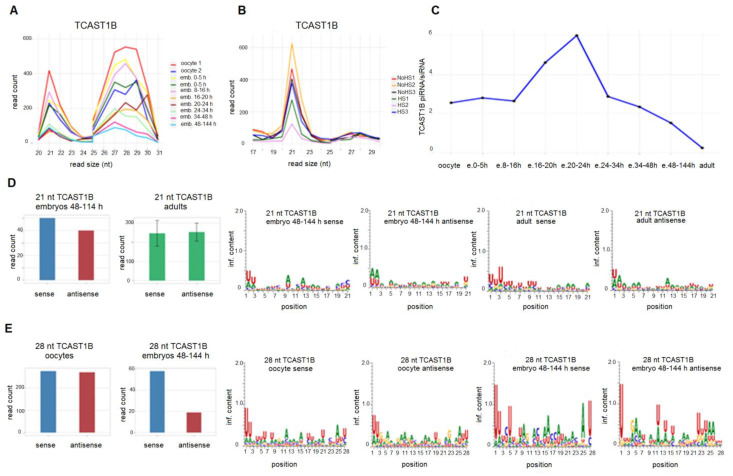
Size and count distribution of reads mapped to TCAST1B satellite in *T. castaneum* oocyte and embryonic small RNA libraries (**A**) and in small RNA libraries of adults kept at normal temperature (NHS), as well as adults subjected to heat stress (HS) (**B**). Ratio between TCAST1B piRNA (26–30 nt) and TCAST1B siRNA (21–22 nt) reads during development (**C**). Number of 21 nt reads mapped on the sense and antisense strand of TCAST1B in embryos (48–144 h) and adults as well as the sequence logos for sense and antisense 21 nt reads (**D**). Number of 28 nt reads mapped on sense and antisense strand of TCAST1B in oocytes and embryos (48–144 h), as well as the sequence logos for sense and antisense 28 nt reads (**E**).

Small RNA reads, which map to the TCAST1 satellite DNA, in particular to the subfamiliy TCAST1B, is comprised of two groups based on their size in all embryonic stages: a minor group encompasses reads corresponding to 20–23 nt with a peak at 21 nt, while a major group contains reads from 26–31 nt, peaking at 28–29 nt, depending on the embryonic stage (Figure 2A). Mapping the small RNA reads obtained from *T. castaneum* control adults as well as adults subjected to HS to TCAST1B revealed a peaking of reads at 21 nt, while out of the 20–22 nt size range, a low number of reads is mapped without a significant peak (Figure 2B). According to the RNA size, the 20–22 nt group could correspond to TCAST1 siRNAs, while the 26–31 nt group could represent TCAST1 piRNAs. TCAST1 piRNA/siRNA ratios differ during embryogenesis in oocytes and early embryos before the onset of zygotic transcription (0–5 h) and an initial population of maternally deposited TCAST1-derived piRNAs and a smaller amount of siRNAs is present with a piRNA/siRNA ratio of 2.5, which remains constant throughout the transcriptionally active early blastoderm (8–16 h). During differentiating blastoderm (16–20 h) and gastrulation (20–24 h), the increase in TCAST1 piRNA/siRNA ratio reaches a maximum level of 6 at 20–24 h embryos. Following gastrulation, the TCAST1 piRNA/siRNA ratio decreases to 1.6 at 2–6 days embryos, and further drops to 0.2 in adults and remains constant after heat stress (Figure 2C).

TCAST1-derived 21–22 nt RNAs map to both DNA strands and their quantification shows a very similar amount of reads deriving from sense, as well as antisense strand during embryogenesis and in adults as well as upon heat stress (Figure 2D; Supplementary Materials Figure S1). This suggests their origin from long double stranded transcripts, which is together with their size of 21–22 nt characteristics of siRNAs. Nucleotide bias analysis of TCAST1 siRNAs from sense and antisense strand reveals, with the exception of a weak preference for U at the first position, no significant enrichment of nucleotides at any position during embryogenesis as well as in adults and upon HS (Figure 2D; Supplementary Materials Figure S1), which is also typical for siRNAs. In contrast, TCAST1 26–31 nt RNAs map to both DNA strands to a similar degree in only oocytes, while throughout embryogenesis, they become strand specific and in late embryos 80% of TCAST1 piRNAs derive from a single dominant strand (Figure 2E; Supplementary Materials Figure S2). In *Drosophila*, germline piRNAs are also produced from both strands, while in somatic cells, piRNAs are derived from a single strand origin [26]. Nucleotide bias analysis of 26–31 nt TCAST1 RNAs reveals significant enrichment of uracil at the first position throughout all developmental stages (Figure 2E; Supplementary Materials Figure S2). Single stranded precursors, the size between 26 and 30 nt, and enrichment of uracil at the first position are all characteristics of piRNAs, and therefore TCAST1 26–31 nt long RNAs could be considered typical piRNAs.

**Figure 3:**
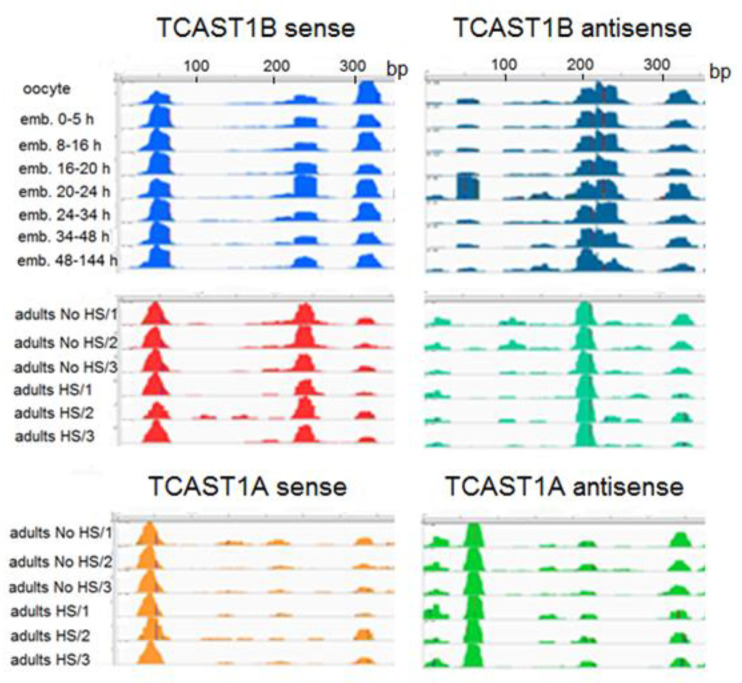
Distribution of piRNA and siRNA reads that map along the sense (**left**) and antisense (**right**) strand of the TCAST1B monomer (360 bp) during embryogenesis, and of siRNAs, which map along the sense (**left**) and antisense (**right**) strand of TCAST1B and TCAST1A monomers, respectively, in adults kept at normal temperature (No HS; in triplicate), as well as adults subjected to heat stress (HS; in triplicate).

Mapping of siRNAs and piRNAs along the TCAST1B satellite sequence reveals their nonuniform distribution. Namely, the majority of them map to several specific regions of the 360 bp monomer, representing the abundant siRNAs and piRNAs, respectively, while the residual TCAST1 siRNAs and piRNAs map on the rest of the sequence (Figure 3). During embryonic development, the abundant piRNAs and siRNAs derive from three prominent regions (positions 30–70 bp, 220–260 bp and 310–340 bp) on the sense strand of the TCAST1B sequence, which are reduced to two regions in adults (Figure 3). On the antisense DNA strand, the abundant piRNAs and siRNAs map to a region between 180–240 bp during embryogenesis, while in adults, abundant TCAST1 siRNAs are derived from the position 180–220 bp and this profile is not changed upon heat stress (Figure 3). For the satellite monomer corresponding to another TCAST1 subfamily, TCAST1A, the single most prominent region on the antisense strand from which abundant siRNAs derive in adults is at position 60–90 bp, and is different from the position of abundant siRNAs at TCAST1B antisense strand (Figure 3). At the sense strand, the single prominent region between 30–70 bp is common in both subfamilies, but the region at 220–260 bp is specific for TCAST1B (Figure 3). This reveals the existence of abundant siRNA, which are specific to the TCAST1 satellite subfamilies A and B, respectively.

**Figure 4:**
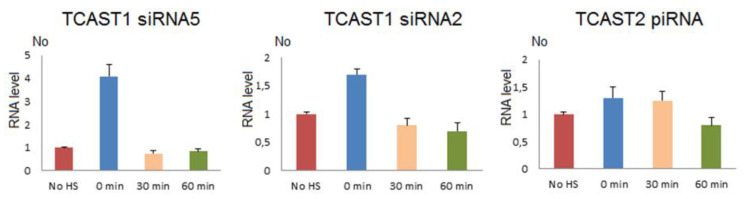
The dynamics of expression of TCAST1 siRNAs 5 and 2, and of TCAST2 piRNA in *T. castaneum* adults under standard conditions (no HS), immediately after 24 h of heat stress at 40 °C (0 min), at 30 min and 60 min of recovery, respectively. Expression is shown in relative term, No values, which are obtained by dividing each No value by No value of control (no HS). Columns show the average of two different RT-qPCR experiments performed in triplicate and error bars represent standard deviations.

To follow the dynamics of expression of TCAST1 siRNAs after HS, we used two TaqMan probes constructed to hybridize with abundant TCAST1 siRNAs, which derive from the 30–75 bp region at the sense strand of both subfamilies TCAST1A and B (Figure 3). The siRNA2 probe encompasses the region 41–61 bp and siRNA5 region 55–75 bp. Adult beetles were subjected to a long term HS and TCAST1 siRNA level, which was measured immediately after HS and at 30 and 60 min of recovery, respectively. The results reveal an increase in TCAST1 siRNAs 4.1x (*p* = 0.005) and 1.7x (*p* = 0.001) using siRNA5 and siRNA2 probes, respectively, immediately after HS, relative to the control, while after 30 min and 60 min of recovery, the levels drop to the level of control (Figure 4). The quantification of TCAST1 siRNAs in RNAseq libraries of *T. castaneum* adults kept at normal temperature and adults subjected to heat stress followed with a recovery time of 60 min revealed no significant difference in the amount of TCAST1 siRNA between the two samples (Supplementary Materials Figure S3), confirming the results obtained by qPCR. The results reveal differences in the dynamics of increase of long TCAST1 transcripts and TCAST1 siRNA upon HS. While the level of TCAST1 transcripts remains increased up to 2 h after HS, the level of TCAST1 siRNAs drops to the basal level more quickly, within 30 min of recovery. A very similar dynamic of small TCAST1 RNA levels upon short term HS was previously observed by Northern blot hybridization [22]. The results suggest the existence of a cellular mechanism that is responsible for the removal of excess satellite-derived siRNAs, which seems to be activated shortly after HS termination. A mechanism that specifically controls the satellite RNA level was recently discovered [27].

### 2.3. Small TCAST2 RNAs during Development and Upon Heat Stress

**Figure 5:**
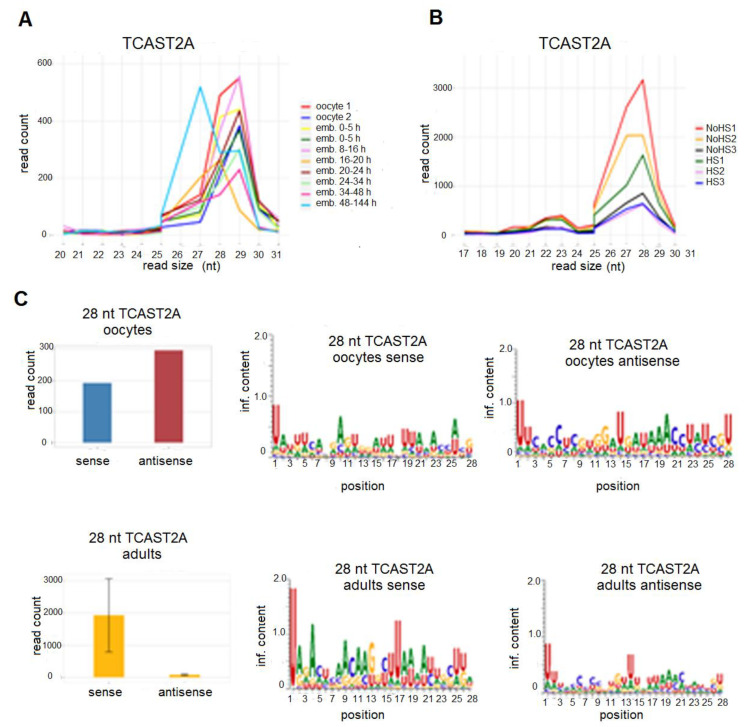
Size and count distribution of reads mapped to the TCAST2A satellite in *T. castaneum* oocyte and embryonic small RNA libraries (**A**) and in small RNA libraries of adults kept at normal temperature (NHS), as well as adults subjected to heat stress (HS) (**B**). 28 nt reads were mapped on the sense and antisense strand of TCAST2A in oocytes and adults, as well as the sequence logos for sense and antisense 28 nt reads (**C**).

Small RNA reads, which map to the TCAST2A satellite DNA, are predominantly in the size range 26–30 nt with a peak at 28–29 nt in most embryonic stages, as well as in control adults and those subjected to HS (Figure 5A,B). Out of this size range, a low number of reads was mapped without a significant peak in embryonic stages and with a small peak at 22–23 nt in adults. According to the size profile of TCAST2A small RNAs, they could be considered piRNAs and their presence in oocytes and early stage embryos gives evidence for maternal deposition. The comparison of a number of reads, which map to the 20–23 nt size range with those mapped to 26–30 nt size range, reveals that 90% of TCAST2-derived small RNAs correspond to piRNAs. TCAST2 piRNAs map to both DNA strand in a similar amount in only oocytes and early embryos, while throughout the embryogenesis, they become strand specific and in late embryos, as well as in adults where most of them derived from a single dominant strand, and the ratio remains constant after HS (Figure 5C; Supplementary Materials Figure S4). Nucleotide bias analysis shows a strong enrichment of uracil at the first position of TCAST2 piRNAs in all embryonic stages and in adults (Figure 5C; Supplementary Materials Figure S4). A size of 26–30 nt, single stranded precursors and enrichment of uracil at the first position are characteristics of piRNAs and are therefore TCAST2-derived small RNAs that can be considered typical piRNAs.

**Figure 6:**
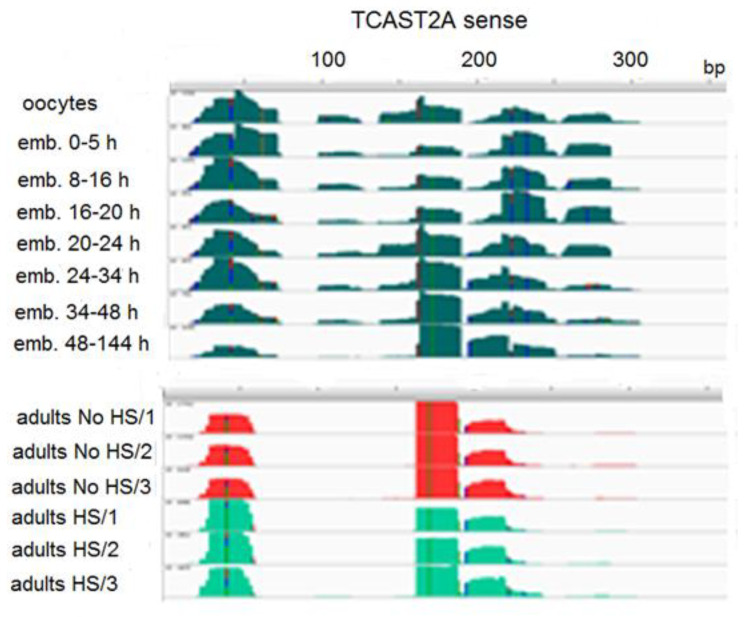
Distribution of TCAST2A piRNA reads along the sense strand of TCAST2A monomer (359 bp) during embryogenesis and in adults kept at normal temperature (No HS; in triplicate), as well as adults subjected to heat stress (HS; in triplicate).

TCAST2 piRNAs derive preferentially from a single DNA strand and their mapping to the dominant sense strand of the 359 bp TCAST2 satellite monomer reveals four regions from which the majority of piRNAs derive during early embryogenesis (Figure 6). The profile of abundant piRNAs is reduced to three major regions (between 30–60 bp, 160–180 bp and 190–220 bp) in late embryos and adults, and is not changed upon heat stress (Figure 6). Generally, the profile of TCAST2 piRNAs in oocytes seems to be characterized by more diverse species of piRNAs, while during embryogenesis and in adults piRNA, the diversity decreases, suggesting specificity of piRNA genesis in the germ line relative to somatic cells. To check the dynamics of TCAST2 piRNAs upon heat stress, we constructed TaqMan probes specific for abundant piRNAs corresponding to 30–60 bp of the dominant strand and followed the expression of the corresponding abundant piRNA in adults immediately after HS and during recovery periods of 30 and 60 min (Figure 4). The qRT-PCR assay reveals a slight increase in the amount of TCAST2 piRNAs upon heat stress which is not statistically significant (*p* > 0.05), but is in accordance with the results obtained for long TCAST2 transcripts (Figure 4; Figure 1B).

### 2.4. H3K9me3 Levels at TCAST2 and TCAST1 Repeats after HS

A positive correlation between the expression of a major TCAST1 satellite DNA after heat stress and the level of silent histone marks H3K9me3/2 at the TCAST1 satellite DNA repeats was previously reported [22,23]. Here, we analyse H3K9me3 levels at TCAST2 satellite repeats after heat stress relative to a control not subjected to HS, as well as on arrays of TCAST1, which served as a positive control.

**Figure 7:**
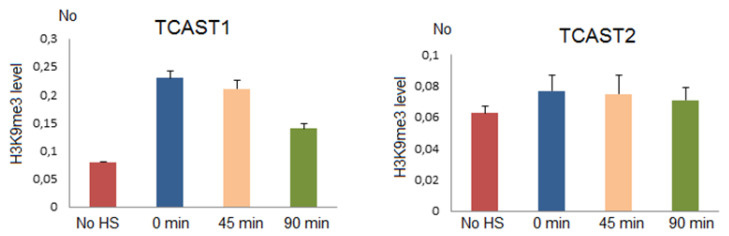
The level of H3K9me3 at TCAST1 and TCAST2 satellite repeats after HS. H3K9me3 levels were measured by ChIP coupled by quantitative real-time PCR at standard conditions (no HS), immediately after 24 h of HS (0 min), at 45 min and 90 min of recovery. No value was normalized using No value of input fraction. Columns show average of three independent experiments and error bars indicate the standard deviations.

Chromatin immunoprecipitation (ChIP) using H3K9me3 antibody was performed on chromatin isolated from adult beetles subjected to 24 h of heat stress at 40 °C immediately after HS and after 45 min and 90 min of recovery at 25 °C, respectively, as well as on chromatin from control beetles. The level of H3K9me3 was quantified by qPCR using primers specific for TCAST2 and TCAST1 repeats, respectively. The results reveal no statistically significant change (*p* > 0.05) of H3K9me3 level at TCAST2 repeats at any point after HS relative to the control, while the level of H3K9me3 at TCAST1 repeats was increased 2.9x (*p* = 0.003), 2.6x (*p* = 0.002) and 1.9x (*p* = 0.004), respectively, immediately after HS, at 45 min and 90 min of recovery (Figure 7). The results support a model according to which the transient increase of satellite transcripts after HS leads to the transient enrichment of silent histone marks at homologous satellite arrays. In the case of the TCAST2 satellite, HS does not significantly induce satellite expression and consequently the level of H3K9me3 remains unchanged.

## 3. Discussion

In the present study, we analysed the expression of two satellite DNAs in the beetle *T. castaneum,* which differ in genomic organization. While the highly abundant TCAST1 satellite DNA is almost exclusively located within (peri)centromeric heterochromatin, the low copy number satellite TCAST2 is, in addition to heterochromatin, also present to a significant level in euchromatic repeat clusters [14,19,24]. Our study reveals that, during development and upon heat stress, the two satellites are characterized by the distinct regulation of transcription and processing of transcripts. TCAST1 satellite transcripts are processed predominantly into piRNAs during embryogenesis and into siRNAs during later development, implying an organization of TCAST1 satellite arrays into two subsets, siRNA- and piRNA-specified arrays, giving rise to TCAST1 siRNAs and piRNA, respectively. It is known that the transcription of dual-strand piRNA clusters in the Drosophila germline is regulated by the chromatin associated Rhino-Deadlock-Cutoff (RDC) complex in which the HP1 paralog Rhino binds to H3K9me3 [28]. It can be proposed that a similar complex is associated specifically to piRNA-specified clusters, which exist in other species, including *Tribolium* and that recognition of piRNA and siRNA genomic targets leads not only to the deposition of H3K9me3, but also to other protein components specific for piRNA or siRNA clusters. Such chromatin associated complexes then define specific chromatin subsets and regulate the transcription as well as biogenesis of siRNAs and piRNAs, respectively.

**Figure 8:**
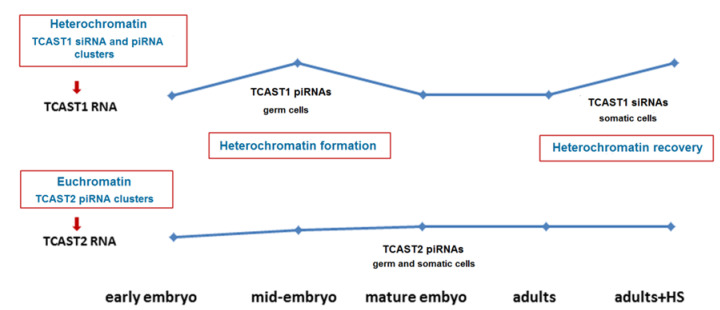
Proposed model that explains the distinct regulation of expression of TCAST1 and TCAST2 satellite DNAs during development and upon heat stress. The burst of TCAST1 transcripts occurs during mid-embriogenesis and the transcripts are predominantly processed into piRNAs, which are assumed to participate in heterochromatin establishment. During later development the processing of TCAST1 transcripts into siRNAs prevails and burst of transcripts upon heat stress (HS) is necessary for heterochromatin recovery. The transcription of TCAST2 RNA proceeds preferentially from euchromatic clusters and is stable during development, not specifically induced during mid-embryogenesis or upon heat stress. TCAST2 piRNAs, which are produced in germ and somatic cells, are assumed to play a role in the establishment and maintenance of euchromatic TCAST2 piRNA clusters, but not in the heterochromatin establishment or recovery. The level of TCAST1 and TCAST2 RNA during development is indicated by blue line.

Differential processing of TCAST1 satellite transcripts into piRNAs during embryogenesis and siRNAs in later developmental stages might be related to distinct roles of TCAST1 piRNAs and siRNAs during development. The burst of TCAST1 transcription in the form of long precursors occur during differentiating blastoderm and gastrulation stages of embryogenesis, coinciding with a change in chromatin condensation [29], and might be associated with the initial establishment of constitutive heterochromatin (Figure 8). After heterochromatin establishment, the level of TCAST1 transcripts decreases and remains constant during larvae, pupae and adult stages [22]. The role of satellite DNA transcripts in the initial establishment of heterochromatin has been described in mouse where a burst of strand-specific transcription of major pericentromeric satellite DNA during embryonic midblastula stage is essential for heterochromatin formation and early development progression [30,31]. Analysis of the small TCAST1 RNA profile in oocytes and early embryos before the onset of zygotic transcription reveals the presence of an initial population of TCAST1-derived small RNAs, mostly piRNAs and in a smaller amount of siRNAs, which are maternally deposited. This implies the generation of TCAST1-derived piRNAs and siRNAs in the germ cells. It was shown in *Drosophila* that transgenerationally, maternally inherited piRNAs enhance the piRNA biogenesis through the instalment of piRNA-specified heterochromatin at homologous genomic regions and by inducing precursor processing [32]. A similar role in the establishment of specific heterochromatin domains can be proposed for the maternally inherited TCAST1 pi- and siRNAs. Throughout the transcriptionally active phases of embryogenesis, the piRNA/siRNA ratio increases up to gastrulation, coinciding with the maximal expression of TCAST1 precursors, as well as with the chromatin condensation [29], suggesting a role for TCAST1 piRNAs in initial heterochromatin formation (Figure 8). Following gastrulation, the TCAST1 piRNA/siRNA ratio decreases, and in later developmental stages TCAST1, siRNAs prevail. According to the TCAST1 piRNA/siRNA ratio during development, it can be proposed that TCAST1 piRNAs biogenesis is characteristic of the germ line, while TCAST1 siRNAs are produced predominantly in somatic cells. In adults, heat stress induces the transcription of TCAST1, and since the increase of TCAST1 transcripts positively correlates with the H3K9me3/2 level at homologous satellite arrays, it is proposed that satellite transcripts reinforce “heterochromatinization” and might be involved in heterochromatin recovery after heat stress [22]. Therefore, while the burst of TCAST1 transcripts and their derived piRNAs in specific stages of embryogenesis might be involved in initial heterochromatin formation, TCAST1 transcripts induced in adults upon heat stress and their derived siRNAs are proposed to play a role in heterochromatin maintenance and its recovery after HS (Figure 8). In favour of this are experiments in *Drosophila*, which showed that the piRNA system plays a guiding role in initial heterochromatin formation during embryogenesis, but has a minor role in the maintenance of heterochromatin during later developmental phases [33]. In addition, an increased level of H3K9me2/3 detected after HS at dispersed TCAST1 satellite repeats correlates with the transient suppression of genes located in their vicinity [23] and points to the role of TCAST1 siRNAs in the modulation of gene expression. We propose that differential processing of TCAST1 transcripts into two forms of small RNAs is enabled by the existence of TCAST1 piRNA and siRNA-specified heterochromatic clusters whose expression is separately regulated and induced either during embryogenesis or during stress response. Such “compartmentalization“ might allow the same satellite DNA to respond specifically to various signals and to participate in different cellular processes.

Transcription of the minor TCAST2 satellite starts in early embryos and gradually increases throughout embryogenesis, but does not show a distinct peak, which is characteristic for TCAST1 transcripts (Figure 8). The absence of a peak during gastrulation indicates that TCAST2 transcription is not affected by heterochromatin condensation. The small TCAST2 RNA profile in oocytes and before the onset of zygotic transcription is characterized by initial maternal deposition of piRNAs preferentially and in a much smaller amount of siRNAs, with the piRNA/siRNA ratio remaining constant during development, which is in contrast to changes in the major TCAST1 piRNA/siRNA ratio. In contrast to the major TCAST1, whose increased transcription is observed upon HS, TCAST2 transcription, as well as the TCAST2 piRNA level, is not significantly affected by HS. It could be proposed that transcription of a major and a minor satellite might be affected by the chromatin state such as heterochromatin, in which TCAST1 is embedded, as well as euchromatin, in which a significant portion of TCAST2 is placed (Figure 8). The influence of chromatin state on the expression of transposon-derived small RNAs during embryogenesis was reported in plants [34]. Heat stress was known to specifically affect heterochromatin by provoking its decondensation and decrease of nucleosome occupancy, resulting in transcription activation of heterochromatic satellite DNAs [35,36,37,38,39]. It is possible that heterochromatin dynamics during embryonic development and upon heat stress influences major TCAST1 satellite transcription, while the TCAST2 satellite transcription, proceeding predominantly from euchromatic clusters, is not significantly affected. Consequently, we did not detect an effect of HS on H3K9me3 levels at minor satellite repeats.

Analysis of a pool of small RNAs, which derive from TCAST repeats, revealed several abundant small RNAs that correspond to specific parts of TCAST sequences. In the case of the abundant TCAST1 siRNAs and piRNAs, they map to almost the same sequence regions in spite of the fact that they derive from distinct precursors that are processed by different enzymes. This might indicate that TCAST1 siRNAs and piRNAs preferentially target the same sequence regions during the process of transcriptional silencing and heterochromatin formation, and that their profile could be defined by the target sequence structure as well as by the efficiency of silencing. It is known that the target RNA secondary structure could be a determinant of siRNA, miRNA and piRNA efficiency [40,41]. While TCAST1 RNA does not exhibit significantly stable secondary structures, it is possible that some sequence sites are particularly accessible for transcriptional silencing and are preferentially targeted by both TCAST1 siRNAs and piRNA. It is interesting that the TCAST1 satellite subfamilies A and B give rise to subfamily-specific abundant siRNAs, which can specifically target each satellite subfamily. This indicates that target sequence specificity might also affect the profile of satellite-derived siRNAs and piRNAs and such specificity could enable distinct regulation of satellite subfamilies by the subfamily specific small RNAs. In addition, there is a sequence bias during piRNA processing characterized by 1U-bias in piRNAs and selection against U within the piRNA body, which restricts the piRNA sequence space and is proposed to be related to their increased specificity and efficiency of silencing [42].

Analysis of the repetitive fraction in many eukaryotic genomes revealed the presence of numerous satellite DNAs, some being species-specific or shared among related species [13,43], while some show deep evolutionary conservation [44,45,46]. Based on the results presented here, satellite DNAs within a genome differ regarding their transcription activation as well as processing of primary transcripts and could represent sequences with distinct functions, being affected by different conditions and involved in various processes. However, it seems that genomic organization and chromatin state might influence satellite DNA expression. In the future, it will be necessary to separately study satellite DNAs present within the genome in order to disclose the interplay between their genomic location, organization and expression regulation during diverse cellular processes.

## 4. Materials and Methods

### 4.1. Tribolium castaneum Beetles

The GA2 strain of *T. castaneum* beetles, which was originally used in the genome sequencing project and deriving from North American wild-type strain collected in Georgia in 1982, were maintained on a mixture of wholegrain flour supplemented with yeast at 25 °C.

### 4.2. RNA Isolation

Total RNA from *T. castaneum* adults used as controls and from adults subjected to 24 h heat stress at 40 °C with a recovery period of 30 min and 1 h, respectively, was isolated using the RNeasy Plus Mini Kit (Qiagen, Hilden, Germany) according to the instructions of the manufacturer. The quality of RNA isolation was checked using an Agilent Bioanalyzer 2100 and RNAs from all samples showed the same level of integrity. Small RNA from *T. castaneum* adults used as controls and from adults subjected to 24 h heat stress at 40 °C and a recovery period of 1 h was prepared using the mirVana miRNA Isolation Kit (Ambion, Waltham, MA, USA) according to the instructions of the manufacturer.

### 4.3. RNA Sequencing and Data Analysis

Total RNA libraries from two biological replicates of *T. castaneum* adults used as controls and adults subjected to 24 h heat stress at 40 °C with recovery periods of 30 min and 1 h, respectively, were prepared using the Illumina TruSeq RNA Sample v2 kit. Sequencing was performed at GeneCore (EMBL, Heidelberg, Germany) by Illumina NextSeq 500 producing 75 bp long paired-end reads and the sequencing data are deposited in the NCBI/bioproject database under accession number PRJNA679427 (https://www.ncbi.nlm.nih.gov/bioproject/679427). RNA sequencing data for *T. castaneum* oocytes, 8–16 h embryos, 16–24 h embryos (gastrulation) and 24–48 h embryos (germband elongation and segmentation) were previously generated [25] and deposited in the GEO database: GSE63770. Sequencing quality was checked with the FastQC program (https://www.bioinformatics.babraham.ac.uk/projects/fastqc/) and Phred values were calculated. Only reads with Phred > 20 were used. Adaptor sequences were removed by the cutadapt program [47]. Reads were mapped on satellite consensus dimer sequences (TCAST1B and TCAST2A) using bowtie2 program [48] and FPKM values were obtained. The program kallisto [49] was also used for the quantification of satellite reads, which were further normalized by DESeq2 package [50].

### 4.4. Small RNA Sequencing Data and Data Analysis

Small RNA sequencing data from three biological replicates of *T. castaneum* adults used as controls and adults subjected to 24 h heat stress at 40 °C and a recovery period of 1 h were prepared using the NEBNext Multiplex Small RNA Library Prep Set. Sequencing was performed at GeneCore (EMBL, Heidelberg, Germany) by Illumina HiSeq2500 producing 50 bp long single-end reads and the sequencing data are deposited in the NCBI/bioproject database under accession number PRJNA679427 (https://www.ncbi.nlm.nih.gov/bioproject/679427). Small RNA sequencing data from oocytes and embryos at 0–5, 8–16, 16–20, 20–24, 24–34, 34–48 and 48–144 h were previously generated [25] and deposited in the GEO database, GSE63770 (https://www.ncbi.nlm.nih.gov/geo/query/acc.cgi?acc=GSE63770). Sequencing quality was checked by FastQC program (https://www.bioinformatics.babraham.ac.uk/projects/fastqc/) and Phred values were calculated. Only reads with Phred > 20 were used. Adaptor sequences were removed by the cutadapt program [47] and reads were shorter than 17 nt and longer than 36 nt were removed by BBDuk. Reads were mapped on satellite consensus dimer sequences using the bowtie2 program [48] and FPKM values were obtained.

### 4.5. Chromatin Immunoprecipitation and Quantitative Real-Time PCR

Adult beetles were frozen and homogenized in Nuclear Isolation buffer (10 mM MOPS; 5 mM KCl; 10 mM EDTA; 0.6% Triton X-100) containing protease inhibitor cocktail Complete Mini (Roche, Basel, Switzerland) and chromatin immunoprecipitation was performed according to the previously published protocol [23]. The following antibodies were used: H3K9me3 (Abcam, Waltham, MA, USA, ab8898) and IgG (Santa Cruz Biotechnology, Santa Cruz, CA, USA, sc2027). Binding of precipitated target was determined by qPCR using the SYBR Green PCR Master mix and the following primers: TCAST1 fw CCATAAGCGAGTTATAGAGTTGG, rev CTTTAGTGACTTTTATGTCTTCTCC and TCAST2 fw TGAGTTTGAGTGTAAATCGGACGC, rev GTTTGATTTATTGCGCCTCGTGG. The same primers were previously used for transcription analysis of TCAST1 and TCAST2 satellite by qPCR assay [18]. The qPCR reactions were done in triplicate, in 50 µL reaction volume with 0.5 µM specific primers, 2X Power SYBR Green PCR Master mix and 30 ng of cDNA. To correct for the differences in sample composition and in the yield of the reverse transcription reaction, ribosomal protein S18 (RPS18) was used for normalization [51,52]. RPS18 was stably expressed without any variation among samples after heat stress. The thermal cycling conditions were as follows: 50 °C for 2 min, 95 °C for 7 min, 95 °C for 15 s and 60 °C for 1 min for 40 cycles, followed by a dissociation stage: 95 °C for 15 s, 60 °C for 1 min, 95 °C for 15 s and 60 °C for 15 s. Amplification specificity was confirmed by dissociation curve analysis and the specificity of the amplified product was additionally tested on agarose gel. Control without a template was included in each run. Post-run data were analysed using LinRegPCR software v.11.1. [53,54]. The software enables calculation of the starting concentration of amplicon (“No value”), which is expressed in arbitrary fluorescence units and is calculated by taking into account PCR efficiency and baseline fluorescence. “No value” determined for each technical replicate was averaged. Averaged “No values” were divided by “No values” of endogenous control for normalization.

### 4.6. Quantitative Real-Time PCR (qPCR) of Small RNAs

Reverse transcription and qPCR analysis of small RNAs was performed using the TaqMan MicroRNA Reverse Transcription Kit (Thermo Fisher Scientific, Walthman, MA, USA) according to the instructions of the manufacturer. cDNA samples were amplified using Applied Biosystem ABI7300 Real-Time PCR System. TaqMan microRNA Assay with TaqMan probes and primers were obtained from Thermo Fisher. The sequences of TaqMan probes were: TCAST1 siRNA2: CUUGUAGGACCAACCAUAAGC; TCAST1 siRNA5 CAUAAGCGAGUUAUAGAGUUG; TCAST2 piRNA: UAAAUCGGACGAUUUAUUUCACGAGUGG. The qPCR reactions were done in triplicate, in a 20 µL reaction with 2X TaqMan Fast Advanced Master Mix and 2X TaqMan microRNA Assay, which contained the TaqMan probe, primers and cDNA. To correct for the differences in sample composition and in the yield of the reverse transcription reaction, tRNA^Lys^ was used for normalization. The expression of tRNA^Lys^ does not change upon heat stress [22] and is constant in all the samples. The statistical significance of difference between RNA expressions was assessed by ANOVA using program R.

## Data Availability

The data presented in this study are openly available in the NCBI/bioproject database, PRJNA679427 (https://www.ncbi.nlm.nih.gov/bioproject/679427) and in the GEO database, GSE63770 (https://www.ncbi.nlm.nih.gov/geo/query/acc.cgi?acc=GSE63770).

## Supplementary Materials

The following are available online at https://www.mdpi.com/1422-0067/22/1/296/s1, Supplementary Materials Figure S1. Number of 21 nt reads mapped on sense and antisense strand of TCAST1B in different embryonic stages and in adults subjected to HS as well as the sequence logos for sense and antisense 21 nt reads. Supplementary Materials Figure S2. Number of 28 nt reads mapped on sense and antisense strand of TCAST1B in different embryonic stages as well as the sequence logos for sense and antisense 28 nt reads. Supplementary Materials Figure S3. Normalized number of reads for 21 nt long TCAST1B siRNAs in control sample (no HS) and after HS followed with 1 h of recovery. Supplementary Materials Figure S4. Number of 28 nt reads mapped on sense and antisense strand of TCAST2A in different embryonic stages and in adults subjected to HS as well as the sequence logos for sense and antisense 28 nt reads.
