# Supplementary material for: Distinct Regulation of the Expression of Satellite DNAs in the Beetle Tribolium castaneum
Source: Int J Mol Sci. 2020 Dec 30;22(1):296. doi: 10.3390/ijms22010296 (PMC7796160; doi:10.3390/ijms22010296)

Suppl. Figure 1. Number of 21 nt reads mapped on sense and antisense strand of TCAST1B in different embryonic stages and in adults subjected to HS as well as the sequence logos for sense and antisense 21 nt reads

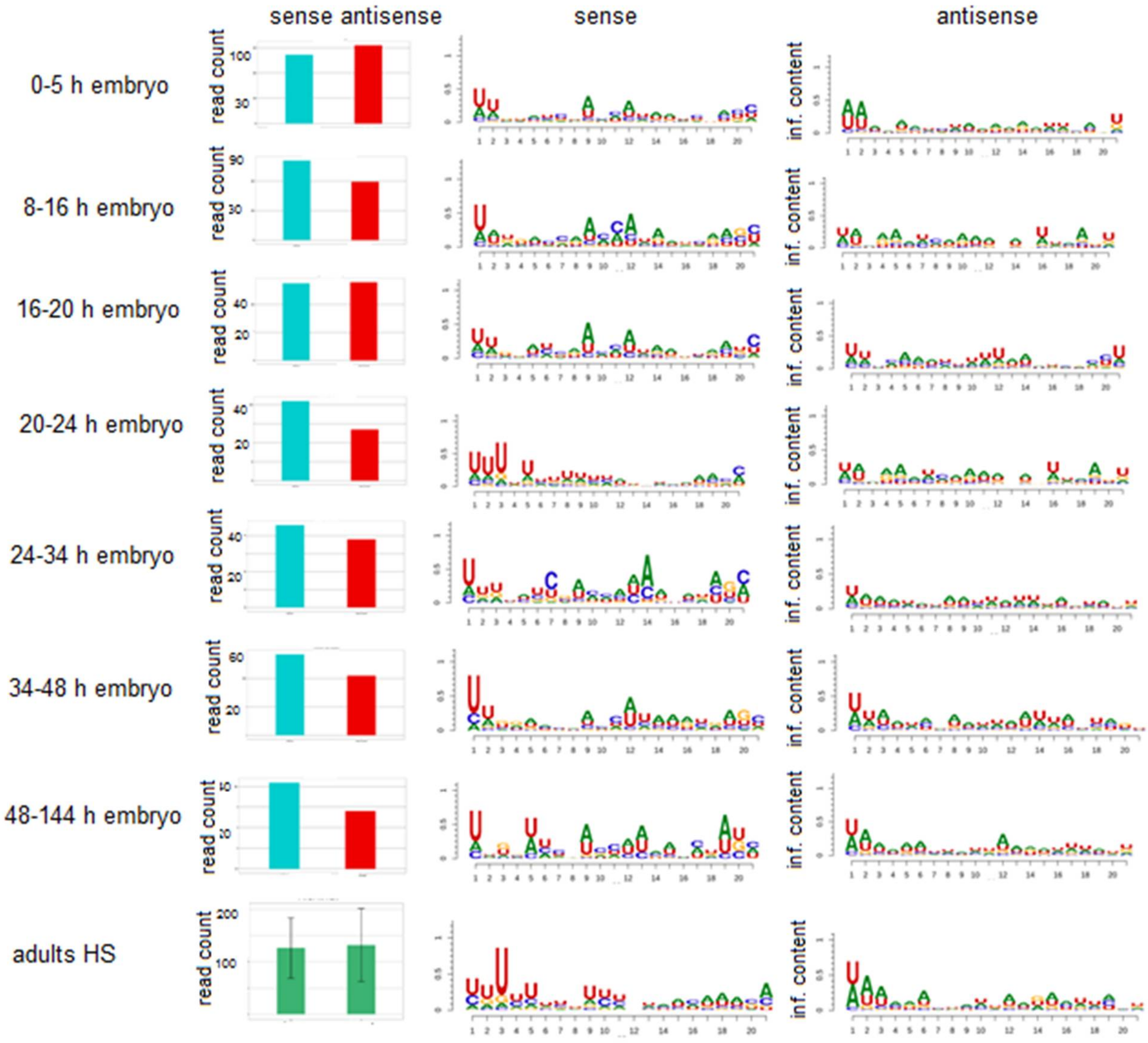

Suppl. Figure 2. Number of 28 nt reads mapped on sense and antisense strand of TCAST1B in different embryonic stages as well as the sequence logos for sense and antisense 28 nt reads.

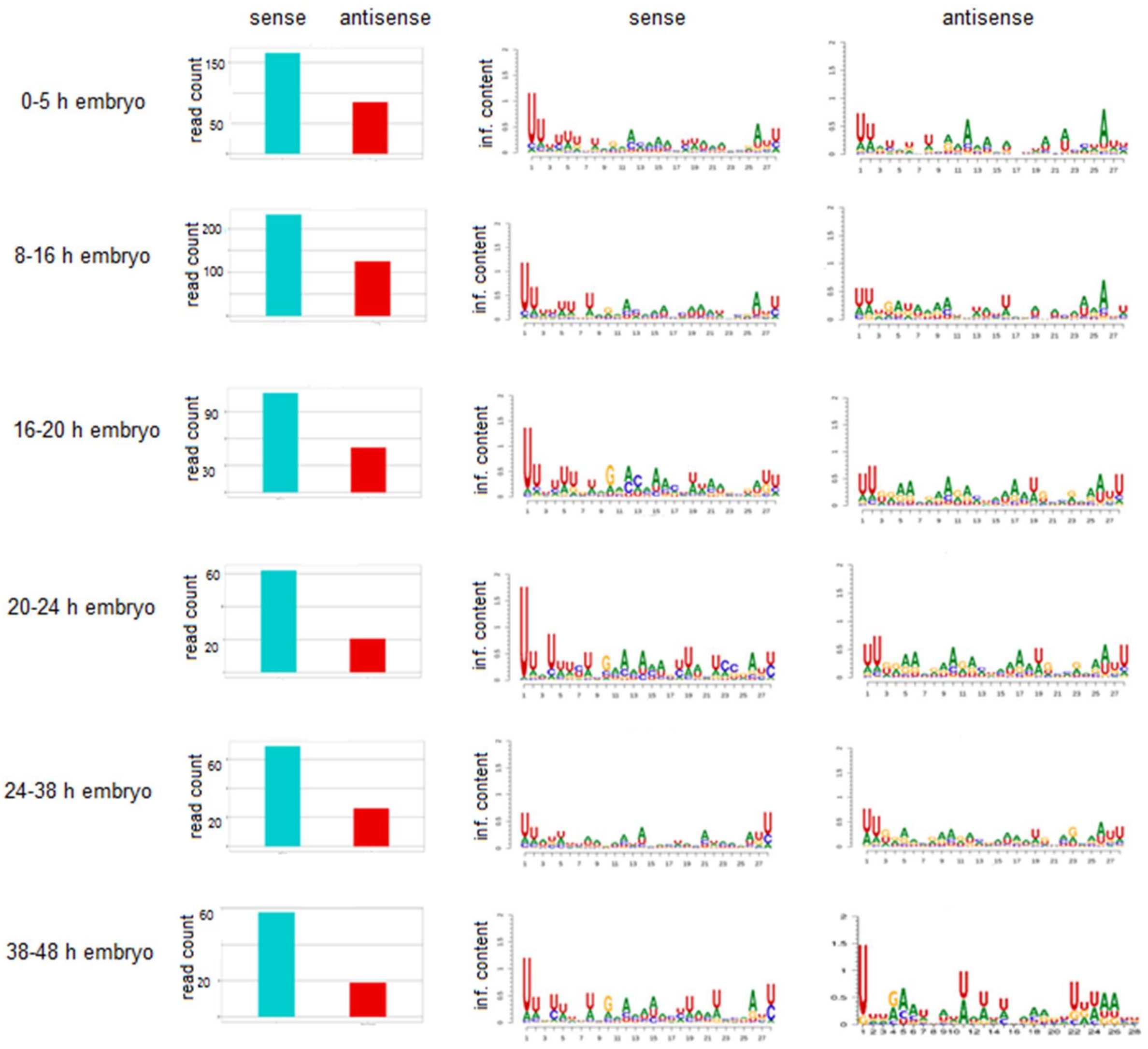

Suppl. Figure 3. Normalized number of reads for 21 nt long TCAST1B siRNAs in control sample (no HS) and after HS followed with 1 h of recovery. Each sample is based on three biological replicates and error bars indicate the standard deviations. Normalization was performed according to the library size.

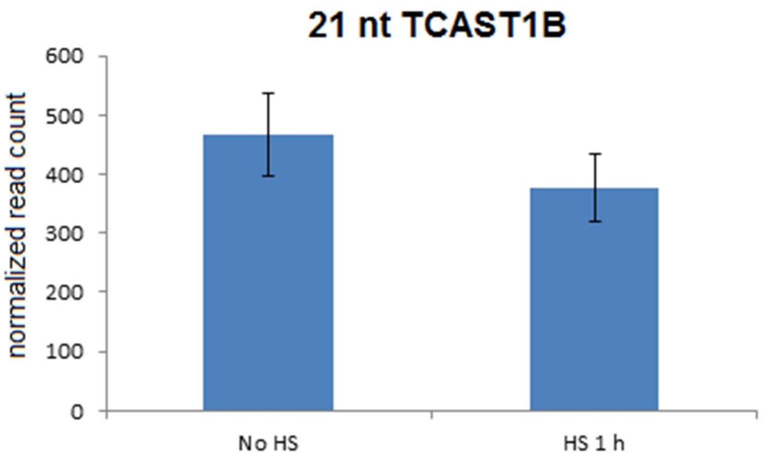

Suppl. Figure 4. Number of 28 nt reads mapped on sense and antisense strand of TCAST2A in different embryonic stages and in adults subjected to HS as well as the sequence logos for sense and antisense 28 nt reads.

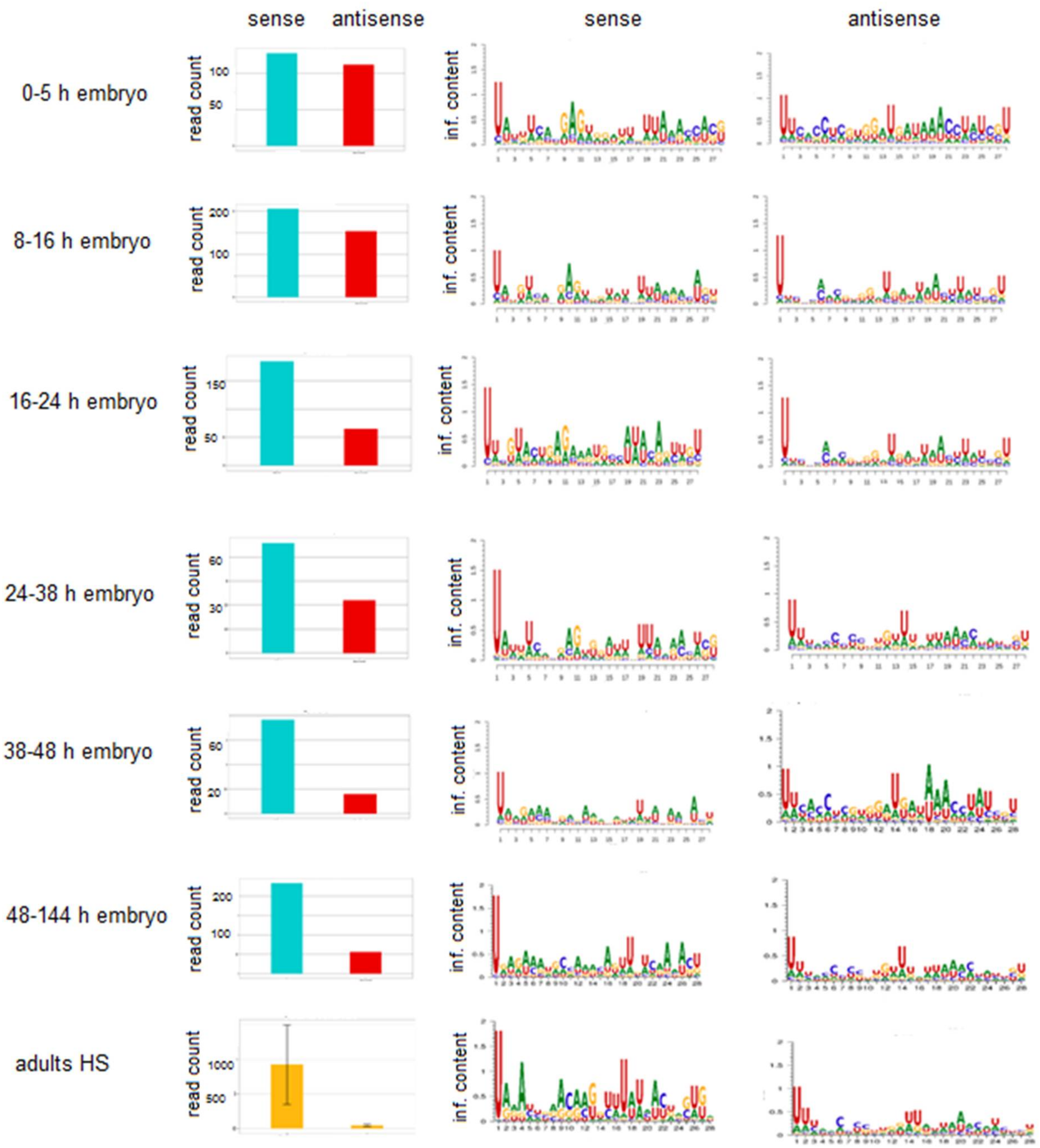

Supplement: Supplementary file 1 [file ijms-22-00296-s001.pdf]
